# Impact of Lower-Limb Muscle Fatigue on Dynamic Postural Control During Stair Descent: A Study Using Stair-Embedded Force Plates

**DOI:** 10.3390/s25175570

**Published:** 2025-09-06

**Authors:** Liangsen Wang, Wenyue Ma, Wenfei Zhu, Qian Xie, Yuliang Sun

**Affiliations:** School of Physical Education, Shaanxi Normal University, Xi’an 710119, China; wlsen13@snnu.edu.cn (L.W.); mawnyue@snnu.edu.cn (W.M.); wzhu@snnu.edu.cn (W.Z.)

**Keywords:** force plate, stair descent, muscle fatigue, lower-limb biomechanics

## Abstract

This study used stair-embedded force plates to investigate the effects of lower-limb muscle fatigue on dynamic postural control during stair descent in young adults. Twenty-five healthy male adults (age = 19.2 ± 1.5 years) were tested for stair descent gait in pre-fatigue and post-fatigue conditions. To induce fatigue, participants performed a sit-to-stand task. The kinematic and kinetic data were collected synchronously, and gait parameters were analyzed. Data were analyzed using one-dimensional statistical parametric mapping (SPM1d) and paired *t*-tests in SPSS. After fatigue, the right knee flexion angle increased significantly across all phases (0–14%, *p* < 0.001; 14–19%, *p* = 0.032; 42–50%, *p* = 0.023; 60–65%, *p* = 0.022; 80–100%, *p* = 0.012). Additionally, the step width widened notably (*p* < 0.001), while the proportion of the swing phase decreased (*p* = 0.030). During the event of right-foot release, the left knee flexion (*p* = 0.005) and ankle dorsiflexion (*p* = 0.001) angle increased significantly, along with a larger left ankle plantarflexion moment (*p* = 0.032). After fatigue, the margin of stability in the anterior–posterior direction (MoS-AP) (*p* = 0.002, *p* = 0.014) and required coefficient of friction (RCOF) (*p* = 0.031, *p* = 0.021) significantly increased at the left-foot release and right-foot release moments. This study demonstrates that lower-limb muscle fatigue increases dynamic instability during stair descent. Participants adopted compensatory strategies, including widening step width, reducing single-support duration, and enhancing ankle plantarflexion to offset knee strength deficits. These adaptations likely reflect central nervous system mechanisms prioritizing stability, highlighting the ankle’s compensatory role as a potential target for joint-specific interventions in fall prevention and rehabilitation. Future studies should investigate diverse populations, varying fatigue levels, and comprehensive neuromuscular indicators.

## 1. Introduction

Stair descent, a highly demanding locomotor task, is common in daily life [1]. Staircases are among the most hazardous locations, both in the workplace and at home, with approximately one out of ten fall accidents occurring on them [2]. These incidents are associated with a significantly higher risk of severe injury or death compared to falls on level ground [3]. This is because stair negotiation, unlike level-ground walking, requires greater lower-limb muscle strength to propel the body upward or downward simultaneously [4]. Studies have shown that, compared with stair ascent, stair descent demands greater balance and postural control and is associated with a higher risk of falls [2]. This requires a coordinated interplay of joint moments and muscle activation to manage impact forces and maintain balance, especially during phases of single-limb support [5]. Therefore, sufficient muscle strength is crucial for preventing falls during stair descent [6,7]. However, muscle fatigue is a frequent occurrence in daily life, which reduces muscle strength and potentially affects motor performance [8]. For example, fatigue occurs after prolonged walking, stair climbing, or other repetitive occupational tasks. Indeed, evidence indicates that approximately one-third of the U.S. workforce engages in substantial physical exertion to meet job demands, often resulting in work-related fatigue [9]. Muscle fatigue is defined as a reduction in the force-generating capacity of the entire neuromuscular system, resulting in a significant decline in muscle strength, impaired neuromuscular control, and reduced motor control performance [8,10]. Extended periods of physical activity can lead to a deterioration of physical capabilities and performance, exacerbating the effects of fatigue, reducing stability, and increasing the risk of accidents, injuries, and falls [8]. Muscle fatigue is a primary task-related risk factor influencing gait [11,12]. Given the heightened biomechanical demands of stair descent compared to level walking, understanding fatigue-induced adaptations in this context is critical for fall prevention. Therefore, investigating the effects of muscle fatigue on stair descent gait is of significant importance.

Although the impact of muscle fatigue on gait has been widely studied in previous research, most studies have focused on level-ground walking or have only analyzed kinematics while ignoring the use of force platforms [13]. Previous studies have demonstrated that fatigue can disrupt joint coordination, delay reflex responses, and reduce dynamic balance during walking [14]. A study found that muscle fatigue may reduce ground dynamic balance, affecting lower-limb kinematics [15]. Conversely, some research has also concluded that after a fatigue intervention, an adult with greater lower-limb strength can still maintain sufficient strength reserves for submaximal-intensity activities, such as walking [16]. From a kinematic perspective, a study found that lower-limb muscular fatigue compromised stair gait during descent [17]. However, few studies have investigated stair gait biomechanics before and after fatigue, particularly utilizing force platforms to quantify kinetic parameters. In addition, most existing studies have primarily used the center of mass (CoM) or center of pressure (CoP) to evaluate balance control. However, the margin of stability in the anterior–posterior direction (MoS-AP) incorporates both CoM velocity and CoP position, providing a more comprehensive measure of dynamic stability [18], while required coefficient of friction (RCOF) reflects the frictional requirements at the foot–ground interface and the associated risk of falling [19,20].

Therefore, this study used stair-embedded force plates to collect kinematic and kinetic data simultaneously and to calculate MoS-AP and RCOF, aiming to investigate the effects of lower-limb muscle fatigue on dynamic postural control during stair descent in young adults. Similar to previous fatigue protocols, we selected multi-joint muscular fatigue of the lower limbs to simulate more realistic scenarios of real-life muscle fatigue [17]. Unlike traditional zero-dimensional (0D) analyses that may overlook time-dependent changes, this study used SPM to analyze stair descent gait, preserving the entire continuous waveform and providing a more sensitive assessment of biomechanical differences [21]. We hypothesize that muscle fatigue will impair dynamic stability during stair descent, altering lower-limb joint kinematics and kinetics. Specifically, fatigue will lead to (1) decreased dynamic stability, as evidenced by increased MoS-AP and RCOF values measured by force platforms, and (2) increased knee flexion and greater ankle plantarflexion as compensatory strategies to counteract reduced quadriceps control.

## 2. Materials and Methods

### 2.1. Participants

This study recruited 25 male participants (age = 19.2 ± 1.5 years; height = 1.79 ± 0.06 m; mass = 74.3 ± 6.8 kg). Inclusion criteria included healthy young adults without neurological or musculoskeletal disorders, capable of independently performing stair ascent and descent. Exclusion criteria included (1) neurological or musculoskeletal injuries affecting gait or balance and (2) history of lower-limb fractures. Foot dominance was assessed based on each participant’s preferred foot for kicking a ball, and all participants were found to be right-foot dominant [22]. Participation was voluntary, and informed consent was obtained in accordance with the principles outlined in the Declaration of Helsinki. This study was approved by the Ethics Committee of the School of Physical Education at Shaanxi Normal University (202516041).

### 2.2. Apparatus and Procedures

**Figure 1:**
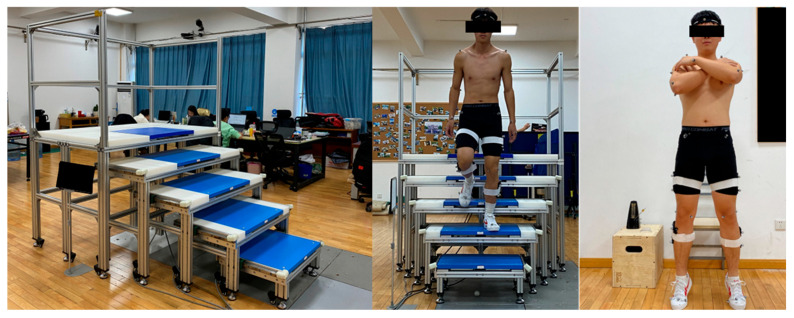
The experiment environment and fatigue protocol.

Our stair-embedded force plate system comprised five steps, with force plates embedded in the first four steps to ensure the collection of complete gait cycles during stair descent. The height between steps is 19 cm, and the width is 30 cm, according to the Chinese national stair standards [23]. Four force plates (Model 9260AA6, Kistler Instrument, Winterthur, Switzerland, 500 × 600 mm, 1000 Hz) were embedded in the staircase’s first, second, third, and fourth steps to collect ground reaction force (GRF) data. Kinematics were obtained using a Qualisys capture system (Oqus 700+, Qualisys AB, Gothenburg, Sweden, 200 Hz) with 10 infrared cameras. Kinematic and kinetic data were collected simultaneously (Figure 1).

All participants wore standardized apparel and footwear throughout the study. Participants first completed a baseline stair gait assessment under non-fatigued conditions, followed by a standardized fatigue intervention 72 h later, with immediate post-fatigue stair gait testing. After warming up with stair-stepping exercises (step-ups on a 20 cm step, 2 min, self-selected cadence) [24], each participant had 58 reflective markers placed at specific anatomical locations [25]. All investigations were conducted under constant conditions, with the temperature controlled at 27 °C and the environment kept quiet during the tests. Each subject completed three valid trials, ensuring the left foot stepped on the fourth step during descent.

### 2.3. Fatigue Protocol

The fatigue protocol targeted the primary muscle groups involved in stair descent, including the quadriceps, gluteal muscles, hamstrings, and gastrocnemius–soleus complex [26]. The fatigue protocol and evaluation methods were referenced from previous studies [27,28]. To induce fatigue, participants performed a sit-to-stand task. A standard chair (42 cm in height, 40 cm in width, and 40 cm in depth) without armrests was used for all participants. The fatigue protocol was terminated when participants reported being unable to continue, failed to maintain the required movement frequency, the Rating of Perceived Exertion (RPE) scale reached 17 or above, or after 30 min had elapsed [27,28]. In addition, conventional sit-to-stand tasks do not adequately fatigue the calf muscles; thus, participants performed the task with simultaneous heel raises to effectively target this muscle group, simulating the fatigue typically experienced in the calf muscles during daily walking (Figure 1).

### 2.4. Data Analysis

The data were extracted as C3D files into Visual 3D (version 6.0, C-Motion, Germantown, MD, USA) for further processing. Kinematic data were filtered using a fourth-order Butterworth low-pass digital filter with a cutoff frequency of 14 Hz, whereas kinetic data were filtered with a cutoff frequency of 50 Hz [29]. An X-Y-Z Cardan sequence defined the order of rotations according to the right-hand rule about the segment coordinate axes. Inverse dynamics were employed to compute sagittal joint torques in the lower limb, with subsequent normalization to the entire gait cycle set at 100% [23,30]. The position data of the CoM were calculated using a 13-segment model with the weighted sum method, where segmental mass proportions and anthropometric parameters were based on Winter’s standardized dataset [30,31]. During the single stance phase, the CoP position in the anterior–posterior (AP) and medial–lateral (ML) directions was determined using the GRFs and torques measured by the force plates at a sampling rate of 1000 Hz [30].

**Figure 2:**
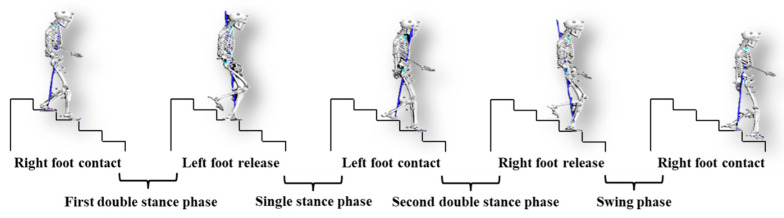

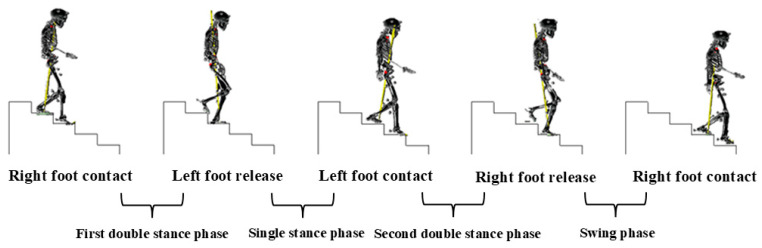
Gait cycle division of the dominant leg (right leg).

Gait events of the foot contact and release were identified with the Visual 3D Software (version 6.0, C-Motion, Germantown, MD, USA) using a threshold of 10N on the vertical ground reaction force [23]. The stair gait cycle of this research begins when the right foot contacts the third step (0% of the gait cycle) and ends when the right foot contacts the first step (100% of the gait cycle). The 5 steps are numbered from bottom to top, from step 1 to step 5. These contact moments define the start and end of the cycle (Figure 2). Gait events include right-foot contact (third step), left-foot release (fourth step), left-foot contact (second step), right-foot release (third step), and right-foot contact (first step). The first four gait events mark the start of the first double-support phase, the single-support phase, the second double-support phase, and the swing phase.

**Figure 3:**
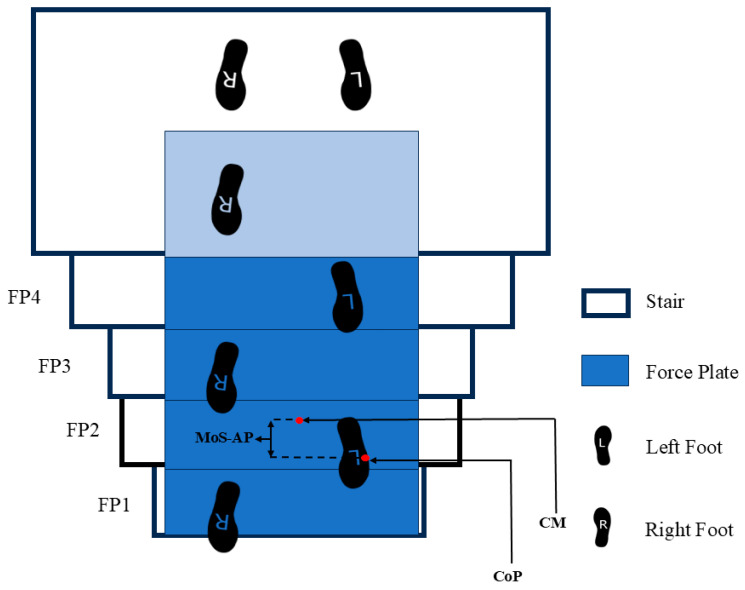
Dynamic stability diagram during stair descent. MoS-AP (margin of stability in the anterior–posterior direction), CoP (center of pressure), and CM (extrapolated center of mass).

To quantify dynamic stability while descending stairs, we applied the inverted pendulum model defined by Hof et al. [18]. In this model, the MoS-AP (Figure 3) is calculated as the difference between the anterior–posterior boundary of the highest base of support (BoS) and the extrapolated center of mass (CM). Given that foot placement and step length, and consequently, the position of the CoP under the foot, are constrained by the staircase geometry, the anterior boundary of the BoS was defined by the edge of the stair [32].

$$CM=pCoM+\frac{vCoM}{\sqrt{g/l}}$$

MoS = BoS − CM.

The BoS represents the maximum support boundary, denoted by CoP in this study. CM is the projection of the center of mass position on the horizontal plane, influenced by velocity. pCoM and vCoM represent the position and velocity of the CoM in the AP direction, respectively; g is gravitational acceleration, and l is the perpendicular distance from the CoM to the ankle joint midpoint. In the sagittal plane, positive values indicate backwards CoM velocity; in the coronal plane, positive values indicate outward CoM velocity. As MoS-AP increases, the distance between CM and CoP increases, reducing stability [33]. The heel position, representing the posterior boundary of the BoS, was determined from reflective markers placed on the posterior aspect of the calcaneus using the 3D motion capture system.

The RCOF is the peak ratio of the horizontal ground reaction force to the vertical ground reaction force. It serves as an indicator of slip risk, which is the leading external factor contributing to postural instability and falls. The RCOF is computed separately during the single-support and swing phases [20]. *F_AP_* = anterior–posterior GRF, *F_ML_* = medial–lateral GRF, and *F_V_* = vertical GRF.

$$RCOF=\frac{\sqrt{F_{AP}^{2}+F_{ML}^{2}}}{F_{V}}.$$

We extracted discrete values of MoS-AP and RCOF at two critical gait events for analysis: left-foot release and right-foot release.

### 2.5. Statistical Analysis

Statistical analyses were conducted using SPSS (SPSS Statistics v25, IBM Corp., Armonk, NY, USA) and MATLAB (MATLAB R2018b, The MathWorks Inc., Natick, MA, USA). The analysis compared conditions before and after fatigue. As key variables, our study included joint angles, moments, MoS-AP, and RCOF. All variables passed the Shapiro–Wilk normality test. Using the spm1d toolbox [34], we conducted paired *t*-tests to compare the right lower-limb joint angles/moments during the gait cycle, where effect size measures were computed for each time point and averaged across significant clusters [35]. Additionally, we conducted paired-samples *t*-tests in SPSS to compare both the left lower-limb joint angles/moments during gait events and the MoS-AP and RCOF between pre-fatigue and post-fatigue conditions. Statistical significance was assumed when *p* < 0.05.

## 3. Results

**Figure 4:**
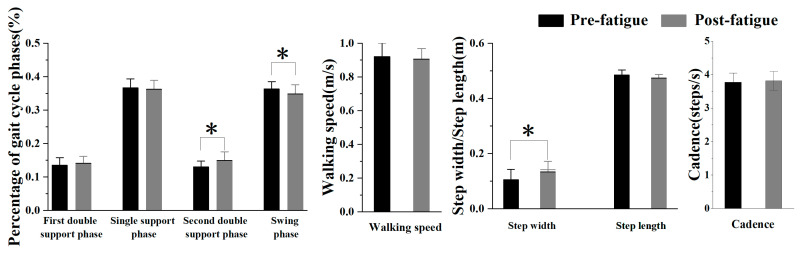
Comparison of spatiotemporal parameters pre- and post-fatigue. *, indicating a statistically significant difference (*p* < 0.05).

All 25 participants completed both the fatigue and non-fatigue conditions, with an average fatigue duration of 12.15 ± 2.43 min. Fatigue resulted in a more extended double-support phase (*p* = 0.005) and a shorter swing phase (*p* = 0.030). Step width increased significantly (*p* < 0.001), with no significant changes in step length, walking speed, and cadence (Figure 4) (Table 1).

**Figure 5:**
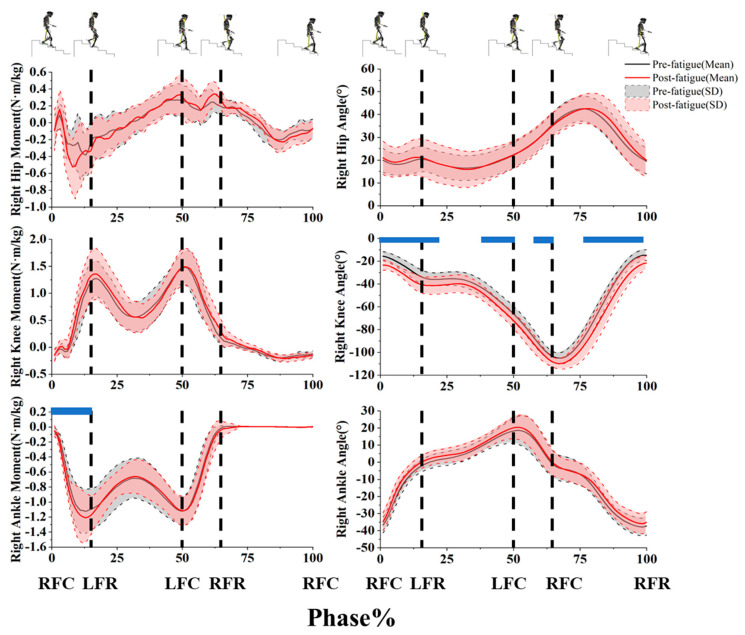
Right lower-limb joint kinetics and kinematics during stair descent derived from SPM1d analysis. The black dashed lines represent gait events: RFC (right-foot contact), LFR (left-foot release), LFC (left-foot contact), and RFR (right-foot release) Blue indicates a statistically significant difference (*p* < 0.05) between pre- and post-fatigue conditions during this phase of the gait cycle. Positive values on the vertical axis represent dorsiflexion, knee extension, and hip flexion, whereas negative values represent plantarflexion, knee flexion, and hip extension.

SPM1D analysis revealed statistically significant differences in lower-limb joint kinematics and kinetics during stair descent. We found that the right knee flexion angle increased significantly during the first double-support phase (0–14%, *p* < 0.001), single-support phase (14–19%, *p* = 0.032; 42–50%, *p* = 0.023), second double-support phase (60–65%, *p* = 0.022), and swing phase (80–100%, *p* = 0.012). The ankle plantarflexion moment increased significantly during the first double-support phase (1–14%, *p* = 0.006) (Figure 5).

Additionally, we analyzed the left lower-limb joint angles and moments at the gait event of right-foot release. After fatigue, an increased knee flexion angle (*p* = 0.005) and an increased ankle dorsiflexion angle (*p* = 0.001) were observed in the fatigue condition versus the no-fatigue condition, and the ankle plantarflexion moment (*p* = 0.032) significantly increased. After fatigue, the MoS-AP (*p* = 0.002, *p* = 0.014) and RCOF (*p* = 0.031, *p* = 0.021) increased dramatically at the left-foot release and right-foot release moments (Table 1).

## 4. Discussion

In this study, we investigate the effects of lower-limb muscle fatigue on dynamic postural control during stair descent in young adults, focusing on lower-limb kinematics and kinetics. Muscle fatigue significantly impaired dynamic stability during stair descent, as reflected by increased MoS-AP and RCOF. Kinematically, fatigue increased knee flexion angles across all gait phases and ankle dorsiflexion angles, widened step width, and reduced the swing phase proportion. Regarding joint kinetics, ankle plantarflexion moments increased in both limbs after fatigue. These findings align with our hypothesis that muscle fatigue impairs dynamic stability during stair descent, while the human body employs a series of compensatory neuromuscular adaptations to maintain postural control.

Descending stairs is more challenging than walking on level ground or ascending stairs [36]. Therefore, fatigued participants may have difficulty controlling their body movements while descending stairs [17]. This phenomenon is validated by the changes in the MoS-AP and the RCOF during stair descent after fatigue observed in our study. Dynamic stability refers to the body’s ability to resist external disturbances and maintain stability during movement [18]. During stair descent, dynamic stability manifests as a series of balance losses and recoveries, particularly in the three-dimensional dynamic process where the CoM constantly changes [32]. Studies have shown that the MoS-AP is typically positive when walking on level ground, indicating a low risk of falling [37]. However, during stair descent, our study found that all participants showed negative MoS-AP values, regardless of fatigue. This is consistent with previous studies [32]. From a mechanical perspective, a negative MoS-AP is necessary and energy-efficient during stair descent, facilitating a smooth transition to the next step [32,38]. Although a negative MoS-AP is typically associated with a higher risk of falling, it does not necessarily indicate an immediate fall. Negative MoS-AP suggests that the individual must take immediate corrective actions to prevent falls [17,39]. The heart center position used in this study is consistent with the dynamic stability concept proposed by Hof [18]. After fatigue, the moment of right-foot release showed a higher RCOF and sagittal plane MoS-AP, indicating increased fall risk and reduced balance control, reflecting compromised postural stability. This change was attributed to muscle fatigue leading to decreased muscle strength, impairing neuromuscular control [12].

Stair descent imposes fundamentally different biomechanical demands than level-ground walking, requiring greater muscle strength to propel the body forward and resist gravitational acceleration [4,36]. This explains why significant kinematic alterations emerge at lower-limb joints when comparing pre-fatigue and post-fatigue conditions during descent [17]. Our study revealed substantial post-fatigue adaptations during stair descent. We found that knee flexion angles increased during all phases, and ankle plantarflexion angles and moments increased during the double-support phase, particularly at single stance. Simultaneously, increased ankle plantarflexion moments in both limbs may compensate for diminished triceps surae strength by requiring greater distal output to sustain propulsion. This suggests an increased reliance on the gastrocnemius and other plantar flexors to enhance push-off power, offsetting diminished proximal joint efficiency [40,41]. In parallel, the widened step width and reduced proportion of swing phase suggest adjustments in spatiotemporal parameters to enhance stability. Studies have demonstrated that an increased step width can expand the BOS, a critical factor in strengthening dynamic stability [42,43,44]. These adaptations align with previous studies reporting greater knee flexion after quadriceps fatigue [45], and that ankle kinematics may adjust through enhanced plantarflexion following fatigue [11,45]. They highlight a joint-specific redistribution of mechanical workload across the lower limb, whereby different joints compensate for reduced function in other segments. Such alterations likely represent coordinated neuromuscular strategies by the central nervous system to preserve postural control and ensure safe forward progression under fatigue [46,47,48,49].

While these findings contrast with reports of reduced joint flexion under limb muscle fatigue protocols [17,50], these discrepancies may stem from differences in experimental design. Multi-joint fatigue may provoke increased leg stiffness [50], resulting in smaller plantarflexion, knee flexion, and hip flexion [17]. This may be due to differences in the fatigue protocols. In contrast, the increased knee flexion observed in our study may reflect a shock absorption strategy. Specifically, the increased knee flexion observed here could reflect an attempt to reduce impact forces by lengthening the time for energy dissipation during landing [51,52]. This strategy could help maintain step-to-step stability even in decreased muscular stiffness or delayed reflexes.

Although some of the statistically significant differences identified were small in magnitude, this could be due to the fatigue level not being sufficiently severe. Participants may not have been required to drastically modify their gait strategies to cope with the demands of stair descent. Similar phenomena have been observed in level-ground walking, where modest fatigue levels can still induce subtle compensatory gait changes. Furthermore, although stair descent requires greater muscular strength than level walking, it may not reach the threshold at which dynamic balance is critically impaired or injury risk substantially increases [15].

Limitations of this study include the following: due to difficulties in recruiting participants and the potential risks of stair descent for older adults, we chose to study healthy young male adults. Future studies should consider including females and older adults with pathologies. Furthermore, this study did not examine the role of upper limb joints, though the upper limbs are crucial for balance. Lastly, future studies should employ varying fatigue protocols to explore the fatigue threshold that disrupts stair descent balance and the compensatory mechanisms of lower-limb joints after fatigue. Moreover, we did not include objective indicators of fatigue intensity, such as electromyographic median frequency or torque decline, which could provide a more precise quantification of fatigue depth. Future studies should employ varying fatigue protocols to explore the threshold at which compensatory mechanisms fail, and to characterize potential nonlinearities in the response of joint kinematics and kinetics to progressive fatigue. Lastly, although the chair and stair heights were fixed, participants had different statures, which may have influenced joint kinematics and kinetics during stair ascent.

## 5. Conclusions

In summary, this study demonstrates that lower-limb muscle fatigue increases dynamic instability during stair descent, which is partially compensated by adaptive postural control mechanisms, including widening step width and reducing single-support duration, while increasing ankle plantarflexion output to compensate for strength deficits in the knee joint. These postural controls likely reflect central nervous system compensatory strategies prioritizing stability to mitigate balance impairments caused by diminished muscular capacity. Although it may appear common sense that fatigue compromises stair descent stability, our study provides quantitative biomechanical evidence to specify how joint kinematics, kinetics, and neuromuscular coordination adapt under localized fatigue. This mechanistic insight is critical because clinical fall prevention and rehabilitation programs require precise targets for intervention rather than general assumptions. For example, identifying the ankle’s compensatory role under knee fatigue may inform joint-specific strengthening or fatigue-resistance training protocols. While the underlying neuromuscular mechanisms remain unclear, future studies should investigate diverse populations, varying fatigue levels, and comprehensive neuromuscular indicators.

## Figures and Tables

**Table 1:** Mean (SD) of joint angle and moment measures during stair descent.

Dependent Measures		Pre-Fatigue	Post-Fatigue	*p*
Spatiotemporal parameters	First double-support phase (% of gait cycle)	0.14 (0.03)	0.14 (0.02)	0.571
	Single-support phase (% of gait cycle)	0.36 (0.03)	0.37 (0.03)	0.251
	Second double-support phase (% of gait cycle)	0.12 (0.02)	0.13 (0.03)	0.005 *
	Swing phase (% of gait cycle)	0.37 (0.02)	0.36 (0.03)	0.030 *
	Cadence (steps/s)	3.77 (0.27)	3.81 (0.28)	0.645
	Walking speed (m/s)	0.92 (0.08)	0.92 (0.10)	0.960
	Step width (m)	0.11 (0.04)	0.12 (0.03)	*p* < 0.001 *
	Step length (m)	0.49 (0.03)	0.48 (0.02)	0.414
Left Hip joint angle (°)	Right-foot release	21.32 (4.36)	22.78 (8.96)	0.312
Left Knee joint angle (°)	Right-foot release	−36.36 (5.24)	−41.11 (6.15)	0.005 *
Left Ankle joint angle (°)	Right-foot release	88.42 (4.55)	91.39 (3.44)	0.001 *
Left Hip joint moment (N·m/kg)	Right-foot release	−0.25 (0.12)	−0.30 (0.20)	0.386
Left Knee joint moment (N·m/kg)	Right-foot release	1.27 (0.33)	1.25 (0.26)	0.796
Left Ankle joint moment (N·m/kg)	Right-foot release	−1.12 (0.21)	−1.24 (0.26)	0.032 *
MoS-AP(m)	Left-foot release	−0.13 (0.04)	−0.23 (0.17)	0.002 *
RCOF	Right-foot release	−0.13 (0.03)	−0.16 (0.02)	0.014 *
	Left-foot release	0.20 (0.05)	0.23 (0.04)	0.031 *
	Right-foot release	0.15 (0.02)	0.17 (0.02)	0.021 *

‘*’ indicates statistical significance. Positive values on the vertical axis represent dorsiflexion, knee extension, and hip flexion, whereas negative values represent plantarflexion, knee flexion, and hip extension.

## Data Availability

The data presented in this study are available on request from the corresponding author. The data are not publicly available due to confidentiality reasons.
